# Use of sodium dodecyl sulfate to improve tuberculosis sputum smear microscopy

**DOI:** 10.15641/ghi.v2i2.824

**Published:** 2019-11-26

**Authors:** Yeya dit Sadio Sarro, Ousmane Kodio, Alisha Kumar, Bassirou Diarra, Bocar Baya, Seydou Diabate, Bourahima Kone, Fanta Sanogo, Mohamed Tolofoudie, Amadou Somboro, Gagni Coulibaly, Boureima Degoga, Mahamadou Kone, Bindongo PP Dembele, Issiaka Camara, Moumine Sanogo, Antieme CG Togo, Nadie Coulibaly, Fatimata Diallo, Etienne Dembele, Brehima Diakite, Seydou Doumbia, Oluwatoyin P Popoola, Souleymane Diallo, Jane Holl, Chad J Achenbach, Robert L. Murphy, Sally McFall, Mamoudou Maiga

**Affiliations:** a University of Sciences, Techniques and Technologies of Bamako (USTTB), Bamako, Mali; b Northwestern University, Illinois, USA; c University of Lagos, Lagos, Nigeria

**Keywords:** tuberculosis, sputum smear microscopy, sodium dodecyl sulfate

## Abstract

Sputum smear microscopy (SSM), the most widely available tool for tuberculosis (TB) detection, has limited performance in paucibacillary patients and requires highly experienced technicians. The objective of this study was to determine whether the addition of sodium dodecyl sulfate (SDS), a detergent that thins sputum, at 4% and 10%, improves the detection of acid-fast bacilli (AFB), the clarity of slides, and the biosafety of the technique. Thirty participants with presumptive TB were enrolled. Three independent, blinded technicians examined the slides. Regular sputum concentrated AFB smear and sputum culture were used as standard control methods. Sputum culture was also performed before and after 10% SDS addition for safety analysis. We found that neither SSM with SDS 4% nor SSM with SDS 10% improved the test’s performance. However, slides with 4% and 10% SDS, compared with slides prepared without SDS, had significantly better clarity scores. The 10% SDS-prepared sputum samples were all culture negative. While adding SDS detergent does not improve the performance of SSM slides, it does improve the clarity and biosafety. Where experienced technicians are scarce, especially in low resource settings, use of SDS may enhance the ease of slide reading in sputum smear microscopy.

## Introduction

Tuberculosis (TB) is one of the leading causes of infectious disease-related morbidity and mortality worldwide (WHO, 2018). In 2017, about 10 million new TB cases and 1.3 million TB related deaths including 0.30 million TB/HIV coinfections were reported. TB is currently the leading cause of death of HIV-infected individuals around the world (WHO, 2018). Around 95% of these TB cases occur in low- and middle-income countries (LMICs) where sputum smear microscopy (SSM) is often the only available TB diagnostic tool because of its relative low-cost, simplicity, and minimum equipment requirement. However, SSM has limited sensitivity compared with sputum culture and requires highly experienced technicians to ensure accurate examination and careful handling to mitigate infection contagion risks (Cattamanchi et al., 2010; Desikan, 2013). Worldwide, many pulmonary TB cases still do not have any bacteriological confirmation and are empirically treated, although this approach, based on symptoms and radiological lesions, has a sensitivity of 61% and specificity of 69% for TB patients, and only 50% sensitivity for HIV co-infected cases (Theron et al., 2014). Molecular assays such as Xpert MTB/RIF™ are overcoming some of the diagnostic limitations of SSM, but they remain expensive and are not available in many LMIC settings.

Although widely used, SSM performs poorly for paucibacillary conditions, such as extra-pulmonary TB, children with TB, and in HIV/TB coinfections (Gebre et al., 1995; Desikan, 2013). Sensitivity is especially poor when the bacterial load in specimens is less than 10,000 bacteria/mL, which represents nearly half of TB cases (Desikan, 2013). In addition, performance and technician safety are largely dependent on experience in examining SSM slides and ability to safely handle the highly infectious samples (Farnia et al., 2002). Sputum samples from TB infected individuals are a viscous mixture, making SSM slides difficult to prepare, examine, and interpret (Angeby et al., 2000; Farnia et al., 2002; Allen, Nicol & Tow, 2016). Bacteria are often unequally distributed in sputum and tend to cluster together, creating a non-uniform sample for SSM slide preparation. Viscosity of the sputum sample can also result in difficulty to identify acid-fast bacilli (AFB) by inexperienced technicians (Allen, Nicol & Tow, 2016).

More recent TB molecular assays use N-acetyl-L-cysteine (NALC) and sodium dodecyl sulfate (SDS) to digest, thin, and homogenize sputum samples before analysis (Verma et al., 2013). SDS is a low-cost detergent that thins sputum in a sample and creates a more uniform distribution of bacteria with better homogenization. These changes to a sputum sample may improve the clarity of SSM slides and improve the detection of AFB (Tacquet and Tison, 1961; Pichula et al., 1981; Hoyer & Jensen, 2004; Allen, Nicol & Tow, 2016). SDS may also inactivate *M. tuberculosis*, thus, potentially protecting laboratory technicians from infectious contagion during the preparation and examination of SSM slides. It has to be noted that regular TB SSM with Ziehl–Neelsen (ZN) and Auramine/Rhodamine (A/R) staining do not discriminate between viable and inactivated bacteria (Dezemon, Muvunyi & Jacob, 2014). We hypothesized that creating a more uniform sample with better homogenization could improve the clarity of SSM slides, the detection of AFB and the level of detection. The aim of this study was to determine whether the addition of SDS to a sputum sample would lead to improved clarity of SSM slides, AFB detection and level of detection, as well as improved inactivation of the bacterium, thereby reducing the risk of infectious contagion and improving the biosafety of the samples.

## Materials and methods

The study protocol was approved by the ethics committee of the Faculty of Medicine Pharmacy and Odontostomatology of the University of Sciences, Technics and Technologies of Bamako (USTTB), approval number: 2017–161CE, Mali and the Institutional Review Board of Northwestern University, approval number: STU00206642. Written informed consent was obtained from all study participants before enrollment.

Between June and November 2018, we conducted a cross-sectional study at the University Clinical Research Center (UCRC) SEREFO Laboratory at USTTB. Patients with clinically presumptive pulmonary TB were screened for enrollment at one of the six Bamako Public Referral Health Centers, each of which has a TB Diagnostic and Treatment Unit (each facility provided their own SSM result for each patient). A total of thirty patients with presumptive TB were enrolled in the study. Each study patient provided one sputum sample (approximately 5mL) in a sterile sputum cup and samples were processed on the day of collection.

All procedures were performed in the Biological Safety Level 3 (BSL3) TB laboratory of the UCRC-SEREFO, which is accredited by the College of American Pathologists (CAP). Each sputum sample (approximately 5 mL) was split into two equal parts: One part was treated with SDS and the other part was used for standard TB diagnostic testing, including concentrated-sputum smear (SS) stained with A/R and sputum culture with Mycobacteria Growth Indicator Tube (BBL MGIT Becton Dickinson, Sparks, Maryland). Samples from 20 presumptive TB cases were enrolled consecutively and had 4% SDS added. Samples from 10 confirmed TB cases that were 1+ or 2+ AFB positive had 10% SDS added. Each study patient had 10 SS slides without SDS prepared and 10 SSM slides prepared 30 minutes after the addition of SDS. Future studies will evaluate shorter time points. All slides were stained with A/R and examined, using a fluorescent microscope, by three independent technicians who were blinded to the TB status of the specimen.

The SSM were scored according to the number of bacilli seen on the slides, as recommended by the World Health Organization (IUATLD, 2000). Results were therefore recorded as follows: No AFB = 0; 2–18 AFB/50 field = +1; 4–36 AFB/10 field= +2; and 4–36 AFB/Field = +3. The clarity/readability of the microscopic field of each slide was scored by each technician, using a scale from 1 to 5, with 1 being the poorest clarity and 5 being the best clarity. Intra-reader and inter-reader variability were calculated, based on AFB scores.

Data were processed and analyzed in JMP®, version 5.0.1. The graph was generated by GraphPad Prism 8 version 8.1.0. Mean readability/clarity scores were plotted and compared using the Mann Whitney test. The Wilcoxon pair test was used to compare averages. P values less than 0.05 were considered significant.

## Results

**Figure 1.**
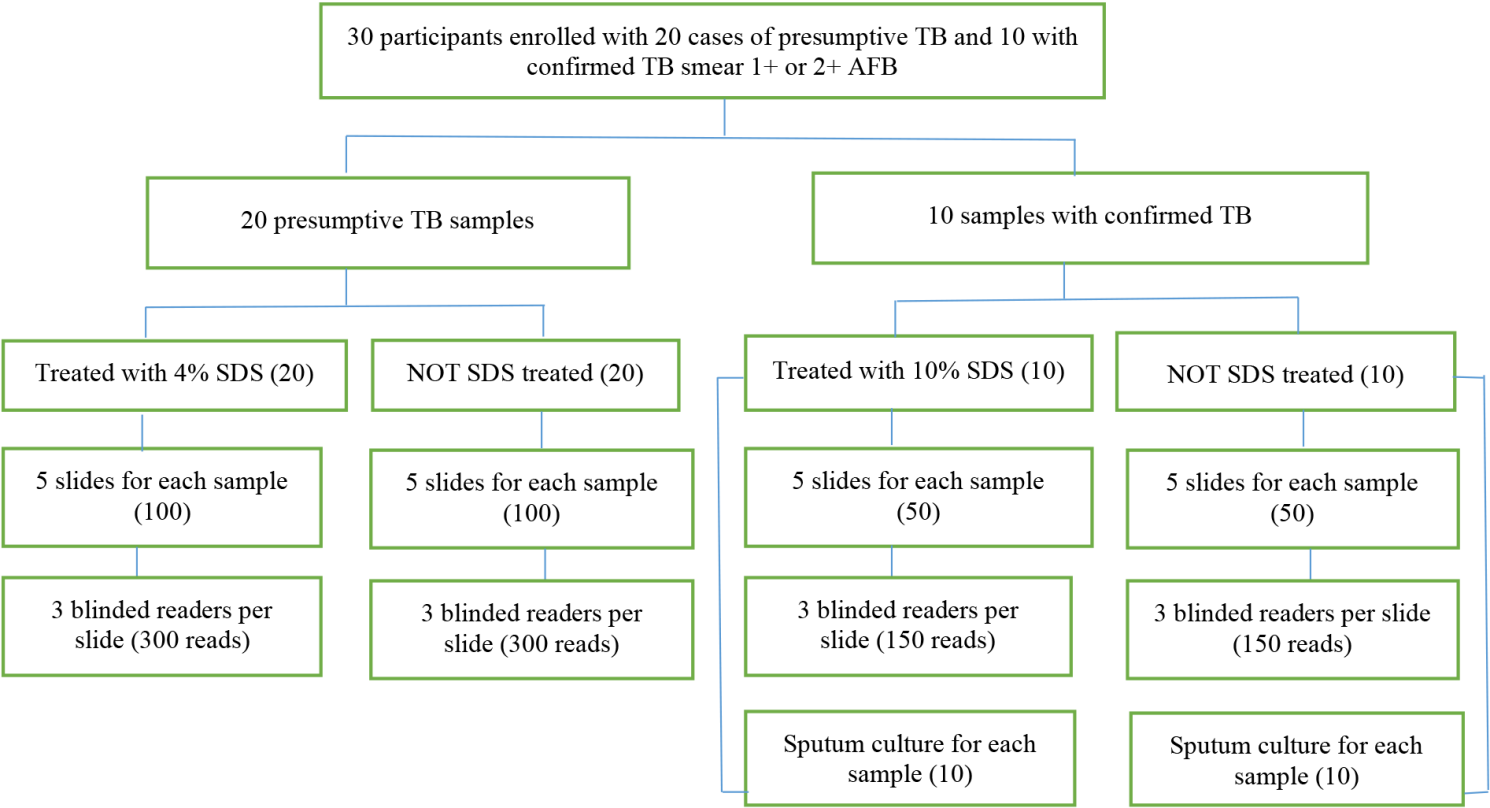
Participants enrolled and laboratory tests performed. SDS=sodium dodecyl sulfate; AFB= acid-fast bacilli.

Of the 30 study participants enrolled, 11 (36%) were female and the mean age was 37.4±15.9 years. All participants were from Mali and 15 (50%) were HIV seropositive. All 30 patients were presumptive or confirmed TB cases. A total of 900 SSM slides were prepared and examined: 450 SSM slides without SDS and 450 SSM slides with SDS (300 with 4% SDS and 150 with 10% SDS). On routine SSM examination, 4 (13%) were 3+, 11 (36%) were 1+ or 2+, and 15 (50%) were smear negative. The participants enrolled and tests performed are shown in Figure 1.

### AFB detection in sputum samples

Detection of AFB and level of detection did not differ between SSM slides prepared with and without SDS, whether 4% or 10%, compared with sputum culture. Variability of AFB detection and level of detection by the three independent and blinded technicians was comparable between SSM slides with and without SDS (Table 2). However, technicians reported a tendency toward better staining quality and somewhat improved homogenization of samples prepared with SDS. Although all SSM slides prepared with sputum with added 10% SDS were only AFB 1+ or 2+, neither AFB detection nor level of AFB detection improved compared to SS slides without SDS (Tables 1 and 2). These slides were read by experienced technicians so that the increase in readability may not have contributed to their ability to achieve consistent results.

### Sputum smear slide readability/clarity

**Figure 2.**
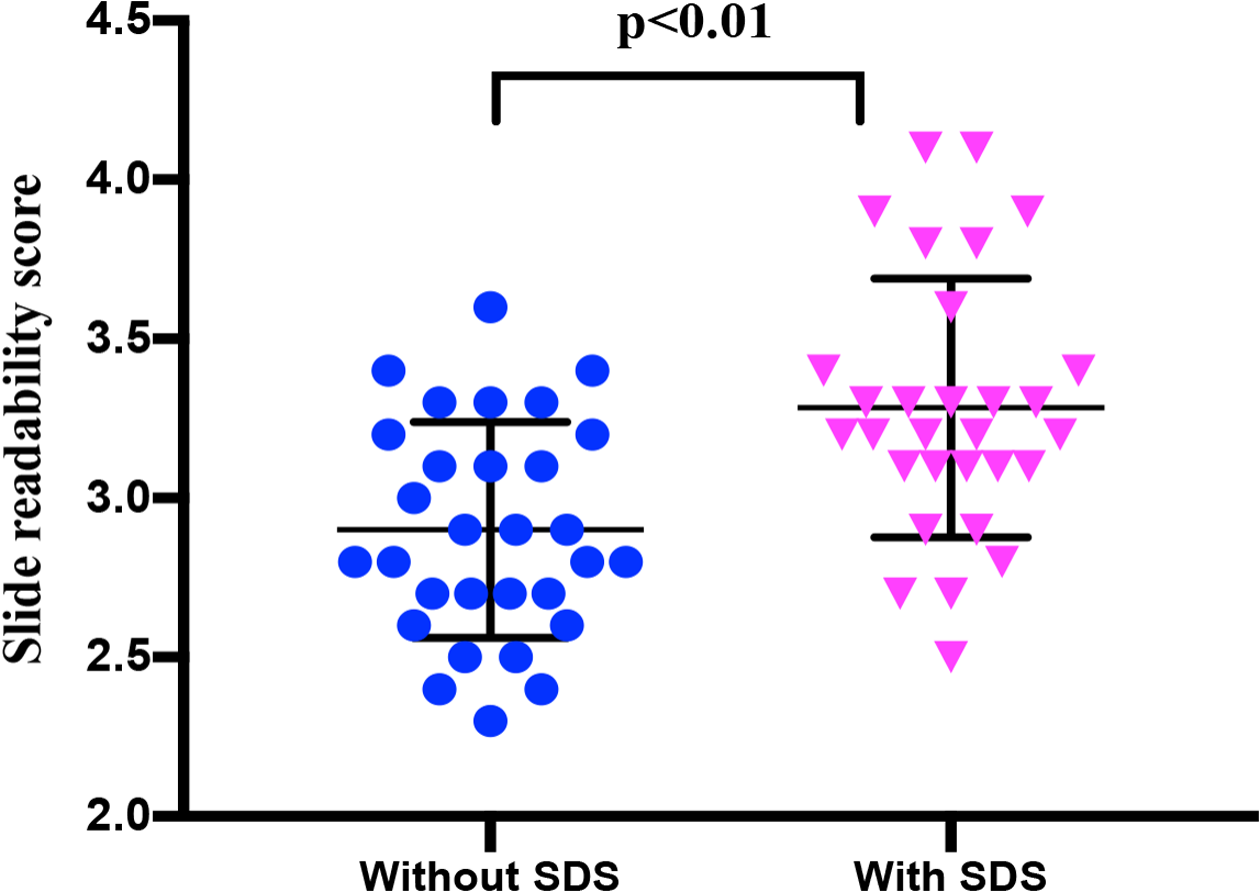
Slide readability/clarity scores with and without sodium dodecyl sulfate (4% and 10% combined). The Mann Whitney test showed statistically significant differences between scores.

**Figure 3.**
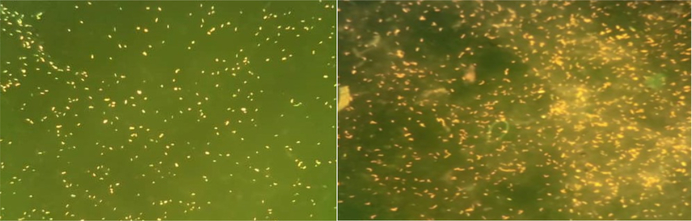
Images of sputum smears with (left) and without (right) 10% sodium dodecyl sulfate.

As shown in Table 1, each technician was asked to score the clarity of each slide. Samples prepared with 10% SDS had a significantly higher clarity score (p<0.01, Mann Whitney test) compared with slides from samples that were not SDS treated (Figures 2 and 3). Slides made with SDS-treated sputum had clearer background with little or no mucus visible. Stained bacilli were easy to find and count (Figure 3). However, slides made with untreated samples had a viscous mucus background, which reduced slide clarity. As a result, the SDS-treated slides had better clarity scores with average scores without SDS and with 4% SDS of 2.94 vs 3.36 (on a scale of 1-poor and 5-excellent) respectively. Average scores without SDS and with 10% SDS were 2.83 and 3.13, respectively (Table 1 and Figure 2). The clarity score was similar in 4 and 10% SDS.

### Sputum smear biosafety

Sputum samples from 10 patients with and without the addition of 10% SDS were cultured. We found that all samples without SDS were culture positive whereas all samples with 10% SDS didn’t grow, suggesting that 10% SDS deactivates *M. tuberculosis*, thereby reducing the risk of infectious contagion and improving the biosafety of laboratory technicians handling sputum samples for performing SSM.

## Discussion

Where the Xpert MTB/RIF test is not readily available, sputum-smear microscopy remains the main diagnostic or follow-up test for detection of TB despite its low sensitivity relative to sputum culture. We found that the addition of SDS significantly improved the clarity of SSM slides and that the addition of 10% SDS improved the biosafety of the slides, although the addition of 4% and 10% SDS did not improve the performance of AFB detection by experienced technicians in SSM.

SSM plays essential role as a diagnostic tool in LMICs, due to its affordability and availability. However, preparation and examination of SSM slides rely on technician expertise which is a major limiting factor in many places where TB is endemic and the availability of expert technicians may be limited or non-existent. Also, personal protective equipment, such as disposable gloves, gowns, respirators, safe practices and use of detergent are sometimes not available in remote laboratories in low resource settings. Therefore, these findings have implications for less intensive training of technicians and less need for protective equipment without compromising biosafety or reducing accuracy of AFB detection.

The SSM slides were independently examined by three expert and blinded technicians to avoid examiner-related bias and offer a robust study design. The lack of significant difference in performance of AFB detection and level of detection between SS slides prepared with and without SDS detergent was unexpected. We hypothesized that the use of SDS would better distribute bacteria and improve homogenization of sputum samples, thereby improving AFB detection and reducing detection variability by technicians. The high accuracy, even with poor clarity, of both non-SDS and SDS smears may be attributable to the experience of technicians.

Further work is needed to assess whether improved clarity could lead to adequate AFB detection by less expert technicians. In remote, underserved health care facilities in LMICs, improvement in SSM slide clarity could potentially permit the use of less intensively trained and experienced technicians, thereby expanding TB diagnostic capacity. For example, SSM slide examination could potentially become a point-of-care (POC) test with nurses being trained to prepare and examine slides. Few TB patients are able to be diagnosed and to initiate treatment at a single visit. Patients are usually required to return for results and treatment initiation days later, which not only increases the attrition rate for treatment but continues to expose patient contacts to TB infection. Given that SSM detects more than half of TB cases (50–60% sensitivity), immediate treatment initiation could reduce disease transmission and mortality with improved treatment rates. In fact, modelling studies have estimated that same-day tuberculosis diagnostics and, therefore, earlier treatment initiation, reduces mortality by up to 35% (Keeler et al., 2006).

The deactivation of *M. tuberculosis* in sputum samples with the addition of 10% SDS detergent greatly enhances the biosafety for those handling the samples for TB diagnosis. This finding has important implications for LMICs where the number of highly trained technicians and the availability of necessary protective equipment to prepare SSM slides are limited: (1) personnel could be trained to a lower level of expertise to adequately detect AFB on SSM slides with higher clarity or (2) higher clarity slides could be read using automated, computerized SSM slide microscopy. Our team at the Northwestern University Center for Innovation in Global Technologies is already working on developing and evaluating an automated system to perform computerized expert-free reading of TB SSM for potential POC testing, as suggested by (García-Basteiro et al., 2018).

The key limitations of this study are (1) the limited sample size and (2) the evaluation of the slides only by expert technicians. A larger, more diverse sample could permit further evaluation of the role of SDS in sputum samples with low bacterial loads, for example in cases of HIV infected patients, and potentially in addressing false negative SSM slides.

## Conclusion

In the present study, while use of SDS has not led improvement in SSM test performance, SDS, as a low-cost detergent, showed potential to improve the clarity and biosafety of SSM. The improved clarity offers an opportunity to achieve adequate diagnostic accuracy by less experienced examiners and the enhanced biosafety may permit point-of-care testing in LMIC settings.

## Figures and Tables

**Table 1.** Sputum smear performance and slide clarity with sodium dodecyl sulfate. SDS=sodium dodecyl sulfate; AFB=acid-fast bacilli.

	SDS	Without SDS average AFB Score	With SDS average AFB Score	Sputum Smear Result from Public Health Center	SEREFO Concentrated Smear Result	Without SDS Average Clarity Score	After SDS Average Clarity Score
**1**	4%	3	3	3	3	3.3	4.1
**2**	4%	0	0	1	1	3.1	3.1
**3**	4%	0	0	0	0	3.6	3.4
**4**	4%	0	0	0	0	3.4	3.8
**5**	4%	0	0	0	0	3.1	3.8
**6**	4%	0	0	0	1	2.9	3.2
**7**	4%	0	0	0	0	3.4	4.1
**8**	4%	0	0	0	0	2.7	3.9
**9**	4%	0	0	0	0	2.4	2.7
**10**	4%	1.10	0.97	1	0	2.7	3.3
**11**	4%	0	0	0	1	2.4	3.3
**12**	4%	0	0	0	0	2.5	2.9
**13**	4%	1.70	1.60	2	1	3.2	3.1
**14**	4%	3	3	2	3	2.7	3.4
**15**	4%	0	0	0	0	2.9	3.2
**16**	4%	0	0.33	0	0	3.2	3.1
**17**	4%	2.90	2.60	3	3	3.3	3.3
**18**	4%	0	0	0	0	2.8	3.3
**19**	4%	0	0	0	0	2.3	3.1
**20**	4%	0	0	0	0	2.8	3.1
**Average, SDS 4%**		**0.59**	**0.58**	**0.6**	**0.65**	**2.94**	**3.36**
**21**	10%	1.33	1	1	3	3.3	3.6
**22**	10%	2.07	1.93	1	3	2.7	2.8
**23**	10%	0.87	0.37	1	1	2.9	3.2
**24**	10%	1.33	1.43	1	3	2.6	2.7
**25**	10%	1.43	1.73	1	3	2.8	3.2
**26**	10%	0.67	0.57	1	1	2.5	2.5
**27**	10%	2.90	2.90	1	3	3.0	3.2
**28**	10%	2.30	2.87	1	3	2.6	3.3
**29**	10%	1.83	1.77	1	3	3.1	3.9
**30**	10%	0.93	0.93	1	3	2.8	2.9
**Average, SDS 10%**		**1.56**	**1.55**	**1**	**2.6**	**2.83**	**3.13**

**Table 2.** Comparison of the mean bacterial load by readers with and without sodium dodecyl sulfate added to samples.

	Mean Bacterial load on SSM	p value with Wilcoxon rank test
**Reader 1**		
With SDS	0.86	0.99
Without SDS	0.88	
Mean Difference	−0.01	
**Reader 2**		
With SDS	0.90	0.92
Without SDS	0.87	
Mean Difference	0.02	
**Reader 3**		
With SDS	0.93	0.90
Without SDS	0.97	
Mean Difference	−0.04	
**All readers together**		
With SDS	0.90	0.97
Without SDS	0.91	
Mean Difference	−0.01	
